# Non-Metastatic Cutaneous Melanoma Induces Chronodisruption in Central and Peripheral Circadian Clocks

**DOI:** 10.3390/ijms19041065

**Published:** 2018-04-03

**Authors:** Leonardo Vinícius Monteiro de Assis, Maria Nathália Moraes, Keila Karoline Magalhães-Marques, Gabriela Sarti Kinker, Sanseray da Silveira Cruz-Machado, Ana Maria de Lauro Castrucci

**Affiliations:** 1 Laboratory of Comparative Physiology of Pigmentation, Department of Physiology, Institute of Biosciences, University of São Paulo, São Paulo 05508-900, Brazil; deassis.leonardo@usp.br (L.V.M.d.A.); nathalia.moraes@usp.br (M.N.M.); keilamagalhaesmarques@gmail.com (K.K.M.-M.); 2 Laboratory of Chronopharmacology, Department of Physiology, Institute of Biosciences, University of São Paulo, São Paulo 05508-900, Brazil; gabriela.kinker@hotmail.com (G.S.K.); sanseray@hotmail.com (S.d.S.C.-M.); 3 Department of Biology, University of Virginia, Charlottesville, VA 22904, USA

**Keywords:** cancer, melanoma, tumor microenvironment, tumor macroenvironment, central and peripheral clocks, chronodisruption

## Abstract

The biological clock has received increasing interest due to its key role in regulating body homeostasis in a time-dependent manner. Cancer development and progression has been linked to a disrupted molecular clock; however, in melanoma, the role of the biological clock is largely unknown. We investigated the effects of the tumor on its micro- (TME) and macro-environments (TMaE) in a non-metastatic melanoma model. C57BL/6J mice were inoculated with murine B16-F10 melanoma cells and 2 weeks later the animals were euthanized every 6 h during 24 h. The presence of a localized tumor significantly impaired the biological clock of tumor-adjacent skin and affected the oscillatory expression of genes involved in light- and thermo-reception, proliferation, melanogenesis, and DNA repair. The expression of tumor molecular clock was significantly reduced compared to healthy skin but still displayed an oscillatory profile. We were able to cluster the affected genes using a human database and distinguish between primary melanoma and healthy skin. The molecular clocks of lungs and liver (common sites of metastasis), and the suprachiasmatic nucleus (SCN) were significantly affected by tumor presence, leading to chronodisruption in each organ. Taken altogether, the presence of non-metastatic melanoma significantly impairs the organism’s biological clocks. We suggest that the clock alterations found in TME and TMaE could impact development, progression, and metastasis of melanoma; thus, making the molecular clock an interesting pharmacological target.

## 1. Introduction

Over the past decades, an overwhelming number of studies clearly established the importance of time in physiology and homeostasis of the organism. The daily pattern of light and temperature over 24 h is detected by a complex and elegant biological clock machinery. Endogenous rhythms are found from bacteria to humans, relying on similar time keeping systems. In mammals, the hypothalamic suprachiasmatic nucleus (SCN) is the central neural oscillator able to respond to cyclic environmental changes of lighting through a retinal melanopsin-dependent process. This photo-pigment, expressed in a subpopulation of retinal ganglion cells [1,2], translates light information into electrical signals, through the isomerization of *cis*- into all-*trans* retinal, with subsequent activation of downstream signaling and release of glutamate and pituitary adenylyl cyclase activating peptide (PACAP) by the retinal hypothalamic tract at the SCN neurons [3]. The SCN then shares this temporal information with several areas of the brain that ultimately control most biological processes. In fact, in a harmonic condition there is only a single time zone within the organism [4,5,6,7].

The molecular basis of the temporal control is an intertwined and complex regulatory system of transcriptional feedback loops involving several genes in the core of the clock molecular machinery [4,6,8,9]. The proteins coded by *Clock* (Circadian Locomotor Output Cycles Kaput) and *Bmal1* (also known as ARNTL, Aryl hydrocarbon receptor nuclear translocator-like protein 1) form heterodimers CLOCK/BMAL1 which migrate to the nucleus and stimulate the transcription of *Per* (Period) and *Cry* (Cryptochrome). After translation, PER/CRY heterodimers are formed and, through phosphorylation by casein kinases, are targeted to the nucleus where they inhibit BMAL1/CLOCK action. This central loop of regulation is fine-tuned by another loop in which BMAL1/CLOCK activates the nuclear receptor subfamily 1, group D, member 1/2 (*Nr1d1*/2 also known as *Rev-Erbα*/*β*) and RAR-related orphan receptor alpha/beta (*Nr1f1*/*2* also known as *RORα*/*β*). REV-ERBα/β and ROR*α/β* compete for the orphan receptor response element (RORE) sequence present in *Bmal1* promoter: REV-ERB*α*/*β* stimulates while ROR*α/β* inhibits *Bmal1* expression. A new cycle of transcription restarts when the inhibitory effect of PER/CRY decreases, mainly due to the degradation of both proteins [8,9,10,11]. The above-mentioned process takes about 24 h to complete and the components of this system have been detected in almost every murine and human cell [8]. In fact, each organ has its own molecular clock, i.e., peripheral clocks, which are influenced by the SCN [9,12]. It is of importance to highlight that CLOCK/BMAL1 also activates the transcription of several clock-controlled genes (CCGs) in a tissue-specific manner, which ultimately contributes to the temporal control of biological processes in the organism. Based on these, clock genes have received increasing interest due to their key role in regulating the body homeostasis [4,6,8,9]. Interestingly, chronodisruption, i.e., loss of internal coherence among the biological clocks, has been linked to the development of cancer [13,14,15,16,17,18,19], metabolic dysfunctions, and psychiatric disorders [4,5,20,21,22].

Melanoma is an aggressive cancer whose incidence has significantly increased over the past decades [23,24]. Melanoma represents 4% of all skin-related cancers but accounts for approximately 80% of all deaths [25]. Although most patients present a localized disease with subsequent surgical excision of the primary tumor, a significant portion of patients develops metastases [26]. In fact, metastatic melanoma is a fatal disease with patients displaying an overall survival of approximately 5 months [27]. Etiologically, melanoma is a multifactorial disease and is associated with chronic environmental and artificial UV exposure, sunburn history in early childhood, reduced skin pigmentation, melanocytic nevi, family history, and genetic susceptibility [23,24]. Interestingly, melatonin, a classic regulator of circadian rhythms [28], is synthesized locally by the skin—a remarkable neuroendocrine system [29]; in this tissue melatonin acts as a protective agent against UV-induced damage [30], but it role as a regulator of the skin molecular clocks is still unclear.

Several genes play a significant role in the development of melanoma. In initial stages, mutation on *BRAF*, *NRAS*, and *NF1* are frequently found while during progression mutations on *TERT*, *CDKN2A*, *ARID1A*, *PTEN*, and *TP53* are observed [31]. In recent years, the landscape of melanoma treatment has significantly changed with the introduction of newer drugs that target BRAF, and its downstream molecule MEK, as well as antibodies that block immune checkpoints such as CTLA-4, PD-1 and its ligand (PD-L1) [32,33,34,35]. In addition to the classic genes related to development and progression of melanoma, as mentioned above clock genes are known to be frequently altered in several types of cancer [14,15,17,18]. Understanding how clock genes are altered in melanoma has become an attractive field of study.

Within this line, our group has shown that the molecular clock of cultured malignant melanocytes is significantly downregulated in comparison to its normal counterpart [16,36,37]. Clinical data show that clock proteins are significantly less expressed in melanoma and in nonmalignant nevus compared to adjacent skin of human samples; in addition, clock genes and proteins correlate with clinicopathological characteristics such as Breslow thickness [38]. An interesting study showed that intra-melanoma tumor dexamethasone injections led to cell cycle arrest with consequent reduction of tumor growth in vivo. This antitumor action is due to dexamethasone-induced activation of the melanoma molecular clock, as the ablation of *Bmal1* resulted in inhibition of the observed glucocorticoid effect [39].

Increasing evidence has implicated the tumor microenvironment (TME) as an important player in tumor development and progression [40,41]. TME contains several cell types such as: immune cells, tumor-associated macrophages and neutrophils, myeloid-derived suppressor cells, fibroblasts, adipocytes, vascular endothelial cells, and lymphatic endothelial cells [41]. This environment not only provides a physical location for all these cells but plays a significant role in development, metastasis, and treatment-related resistance of cancer [40,41,42,43,44].

On the other hand, the crosstalk between the tumor and its macroenvironment (TMaE), i.e., distant organs and tissues, has been less investigated. The best-known example is the cancer-induced cachexia, characterized by progressive loss of muscle mass that cannot be reversed by nutritional support [45,46,47]. This syndrome is mainly due to increased levels of serum cytokines, tumor-derived inflammatory factors, and others secreted by the host in response to the tumor presence [45,46,47]. Other characteristics of TMaE are generalized immune system suppression and altered coagulation capacity [48]. In fact, such alterations in the TMaE are the main source of cancer-related death rather than the effects of tumor growth and metastases themselves [48]. Recently, tumors have also been shown to impact the TMaE through the modulation of molecular clocks. An elegant study demonstrated that a non-metastatic lung adenocarcinoma rewires the time-dependent hepatic metabolism via a pro-inflammatory pathway, with no change in the clock core machinery [49]. In another model, a non-metastatic breast cancer, an altered pattern of clock gene expression was found in liver of the tumor-bearing mice [50].

Our goal in this study was to evaluate whether a non-metastatic melanoma, achieved by subcutaneous inoculation of B16-F10 murine melanoma cells in C57BL/6J mice, affected the machinery of peripheral clocks present in tumor-adjacent skin, lung, and liver (common sites of metastasis), as well as the central SCN oscillator. In addition, we also evaluated the behavior of tissue-specific genes facing the challenge imposed by the tumor.

## 2. Results and Discussion

### 2.1. Effects of Tumor Microenvironment (TME) on Clock and Clock-Controlled Genes of Tumor-Adjacent Skin and Melanoma

Chronodisruption in cancer is often analyzed by comparing the levels of clock genes and/or proteins in single time points between tumor samples, tumor-adjacent and/or healthy tissues; however, to the best of our knowledge, there has not been a study that evaluated the oscillatory process along 24 h of the molecular clock in tumor and tumor-adjacent tissue.

**Figure 1:**
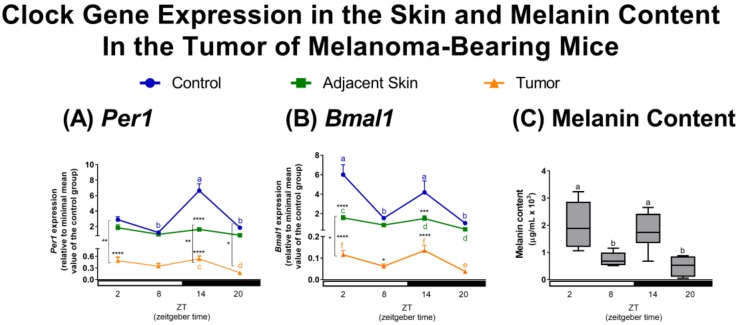
(**A**,**B**) Non-metastatic skin melanoma affects clock gene expression in skin. Expression of (**A**) *Per1*, (**B**) *Bmal1* in control skin, tumor-adjacent skin, and tumor samples. Letters represent significant differences in gene expression within the same group, Two-Way ANOVA followed by Tukey post-test. In (**A**) *a ≠ b*, *p* < 0.01, and *c ≠ d*, *p* < 0.05. In (**B**) *a ≠ b*, *p* < 0.01, *c ≠ d*, *p* < 0.01, and *e ≠ f*, *p* < 0.05. Significant differences among groups were demonstrated by Two-Way ANOVA followed by Bonferroni post-test, and are represented by asterisks having the control skin as reference. Differences between tumor-adjacent skin and tumor samples are indicated by brackets. * *p* < 0.05, ** *p* < 0.01, *** *p* < 0.001, **** *p* < 0.0001. Values are presented as the mean expression (*n* = 3–7) ± SEM of the gene of interest normalized by Rpl37a RNA, relative to the minimal value of skin control group. (**C**) Temporal tumor melanin content. Boxplots show the median, quartiles, maximum, and minimum melanin contents. Melanin content was normalized by total protein. Letters represent statistical temporal differences in melanin content as revealed by One-Way ANOVA followed by Tukey post-test. *a ≠ b*, *p* < 0.05.

Our in vivo analysis of 24 h gene expression showed an oscillatory profile of *Per1* and *Bmal1*, which were in antiphase (Figure 1), a well-established feature of a functional clock [8]. *Per1* levels were decreased at ZT14 (Figure 1A) and *Bmal1* levels at ZT2 and ZT14 (Figure 1B) in tumor-adjacent skin and tumor as compared to control skin. The oscillatory profile of *Per1* was absent in tumor-adjacent skin and very attenuated in tumor samples (Figure 1A), whereas the temporal oscillation of *Bmal1* was sustained, but severely reduced in amplitude, in both tumor-adjacent skin and tumor itself (Figure 1B).

These results indicate that *Per1* and *Bmal1* expression levels are significantly impaired in both tumor-adjacent skin and tumor samples when compared to skin of control, non-inoculated, animals resulting in loss of oscillatory profile of *Per1*. The assumption of “healthiness” of tumor-adjacent skin has resulted in its use as control in skin-related studies [38,51]. In fact, a compelling argument for the use of tumor-adjacent skin is that its morphology is preserved and no sign of malignization is found [51]; however, this traditional view has been recently challenged. Transcriptome analysis comparing heathy, tumor-adjacent tissue, and tumor samples from eight types of tumors, revealed that the tumor-adjacent tissue represents an intermediate state between heathy and tumor tissue [51]. Interestingly, tumor-adjacent is influenced by the pro-inflammatory signals originating from the tumor itself [51]. In fact, our data clearly corroborate this intermediate state of the tumor-adjacent skin. Thus, in addition to the findings reported by Aran and colleagues [51], our findings add another layer of complexity: The disruption of the molecular clock. Therefore, these results warn for further caution in using tumor-adjacent tissue as control.

Since melanogenesis is a clock-controlled process [16,36,52,53], we evaluated the melanin content in tumor samples along 24 h. To the best of our knowledge, we are the first to show a circadian profile of tumor melanin content. Interestingly, the oscillation of melanin content (Figure 1C) showed a similar profile to the one found for *Bmal1* gene (Figure 1B) in the tumor samples: It peaked at ZT2 and ZT14 and had its lowest values at ZT8 and ZT20 (Figure 1C).

We found a positive correlation between *Per1* or *Bmal1* expression with melanin content of tumor samples (Table 1). Previous studies have demonstrated a negative relationship between clock gene expression and melanin content [16,36,53]; however, these studies have used homogeneous melanocyte cell culture while in our study we used the whole skin and tumor samples. In the skin, melanocytes represent only 8% of the epidermal cell population [54] while in the tumor there are several cell types that make up the TME [41]. Therefore, the difference in gene correlation could be explained by the presence of skin or tumor cell types other than pigment cells.

Although the tumor molecular clock was severely downregulated, the oscillatory profile found for melanin content favors the hypothesis that the disrupted tumor clock may still evoke time-dependent local responses. Alternatively, but not exclusively, another hypothesis would be that SCN timing signals or environmental cues feed the tumor clock. Our data could lead to an interesting outcome if proven in patients. It is known that melanin content in melanoma tumors is a prognostic factor since patients harboring tumors with increased melanin content display reduced survival compared to amelanotic melanomas [55]. The presence of melanin in metastatic melanoma is a key factor that contributes to the reduced success of radiotherapy [55]. Accordingly, in vitro studies have provided evidence that inhibiting melanin synthesis improves radiotherapy success in melanoma cell lines [56]. Based on these findings, one could speculate a better outcome of a time-modulated radiotherapy or chemotherapy regimens at the time points where tumors show reduced melanin content; however, this hypothesis needs to be validated in clinical trials.

**Figure 2:**
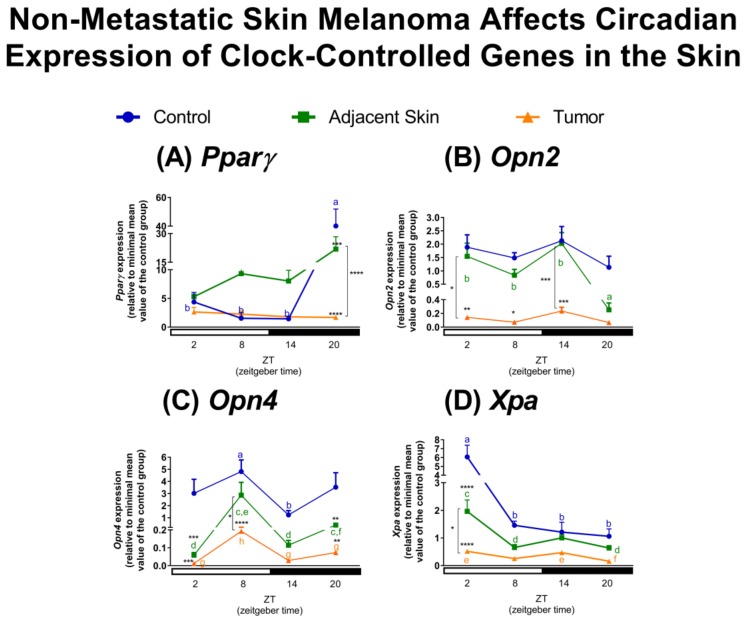
Non-metastatic skin melanoma affects clock-controlled gene expression in skin. Expression of (**A**) *Pparγ*, (**B**) *Opn2*, (**C**) *Opn4* and (**D**) *Xpa* in control skin, tumor-adjacent skin, and tumor samples. Letters represent significant differences in gene expression within the same group, Two-Way ANOVA followed by Tukey post-test. In (**A**) *a ≠ b*, *p* < 0.01. In (**B**) *a ≠ b*, *p* < 0.01. In (**C**) a ≠ b, *p* < 0.05, c ≠ d, *p* < 0.0001, e ≠ f, *p* < 0.05 and g ≠ h, *p* < 0.01. In (**D**) a ≠ b, *p* < 0.001, c ≠ d, *p* < 0.01 and e ≠ f, *p* < 0.05. Significant differences among groups were demonstrated by Two-Way ANOVA followed by Bonferroni post-test, and are represented by asterisks having the control skin as reference. Differences between tumor-adjacent skin and tumor samples are indicated by brackets. * *p* < 0.05, ** *p* < 0.01, *** *p* < 0.001, **** *p* < 0.0001. Values are presented as the mean expression (*n* = 3–7) ± SEM of the gene of interest normalized by Rpl37a RNA, relative to the minimal value of skin control group.

In addition to clock genes, we also evaluated some tissue-specific genes that are involved in proliferation [57,58], detection of light and/or heat [16,59], and DNA repair in tumor-adjacent skin and the tumor itself [60]. Members of the *Ppar* family have been described as direct targets of the CLOCK/BMAL1 dimer through its binding to E-box element present in *Ppar* promoter [61]. *Pparγ* participates in pigmentation and antiproliferative processes in melanocytes and melanoma cells, respectively [57,58]. In our model, *Pparγ* expression was higher at ZT20 in comparison to the remaining ZTs in control skin. No oscillatory profile was found in tumor-adjacent skin or in tumor samples. In the former, due to the overall increase of expression in ZTs 2, 8 and 14 (although not statistically significant), and in the latter due to the dramatic reduction of *Pparγ* transcripts at ZT20 as compared to control skin (Figure 2A). The loss of oscillatory profile of *Pparγ* expression in the skin of tumor-bearing mice may represent important tumorigenic process that contributes to the loss of temporalization of the proliferative process, a common event in cancer [62]. In fact, *Pparγ* expression in tumor-adjacent skin showed a negative correlation with tumor melanin which was lost in tumor samples (Table 1).

The functionality of the photo- and thermo-sensitive systems of the skin is still an ongoing field of studies and their role in pigmentary response and hair follicle growth has been demonstrated [63,64,65,66]. In the skin, OPN2 has been implicated as a sensor of UVA radiation and violet light, participating in pigmentation and differentiation of skin cells [66,67]. In normal and malignant murine melanocytes, we have also demonstrated that OPN2 acts as a UVA radiation sensor [63]. In the present study, *Opn2* did not show a circadian oscillatory profile in control skin or tumor samples; however, in tumor-adjacent skin *Opn2* level was dramatically reduced at night (ZT20) in comparison to the remaining ZTs. In tumor samples *Opn2* expression was significantly reduced in most time points when compared to control and tumor-adjacent skin (Figure 2B). The reduction of *Opn2* transcripts in melanoma tumor in vivo corroborates previous similar findings in cultured melanoma cells [16].

Interestingly, our group detected OPN4, classically found in the retina [1,68], in murine normal and malignant melanocytes [16]. We showed that OPN4 is a UVA radiation sensor [63], and acts in conjunction with OPN2 to mediate the immediate pigmentary process (IPD) [63]. In healthy skin, as well as in tumor-adjacent skin and tumor, *Opn4* expression peaked during the light phase, showing an oscillatory profile which was reduced in amplitude in the tumor (Figure 2C). Since the knowledge of the biological processes regulated by this system in cancer is still scarce, it is hard to grasp the consequences of such reduced gene expression. Nevertheless, higher levels of OPN3 were demonstrated to sensitize hepatocellular carcinoma to 5-fluorouracil. The reduction of OPN3 activates an anti-apoptotic pathway while overexpression of OPN3 inactivates this pathway, thus, favoring chemical-induced cell death [69]. Still in this line, a recent study demonstrated that upon stimulation with blue light, human colon cancer cell lines display decreased viability with increased autophagy, but not apoptosis, compared to non-irradiated cells. Remarkably, all these effects were lost upon knockdown of *OPN3* [70].

Regarding the correlation of opsins and tumor melanin content, we have found that in tumor-adjacent skin and tumor, *Opn2* and *Opn4* correlate positively and negatively, respectively, with melanin content of tumor (Table 1). It is of interest to further investigate the role of opsins and melanin content since OPN2 and OPN4 are UVA sensors and participate in IPD process in melanocytes [63].

Xeroderma Pigmentosum, Complementation Group A (XPA) protein is a DNA repair enzyme, reported to oscillate along 24 h in murine skin [60]. Here, we found that *Xpa* transcripts peaked at ZT2 in control skin, an event that was conserved in tumor-adjacent skin and tumor samples, although with reduced amplitude similarly to other genes analyzed in the skin (Figure 2D). Interestingly, mice exposed to UVB radiation in the morning, when XPA protein levels were low, demonstrated increased skin cancer incidence as compared to mice exposed to the same radiation at night [60]. In our study, *Xpa* transcripts showed higher levels in the light phase and reduced expression in the other ZTs, which is in agreement with previous report of XPA protein peak at ZT10 [60]. Within this line, a recent report demonstrated that an evening cisplatin-based regimen is less toxic than morning treatment in wild type mice, since XPA in the kidney oscillates in a time-dependent manner, leading to more efficient DNA damage removal in the evening compared to the morning. This time-dependent event was lost in *Per1*/*2* KO animals; however, *Per1*/*2* KO mice displayed increased immune system activation which led to increased tumor shrinkage in response to cisplatin in comparison to wild type mice [71]. In our study, *Xpa* in tumor-adjacent skin positively correlates with melanin content while in tumor the correlation was negative (Table 1). This difference could be related to different DNA repair capabilities between both tissues; furthermore, melanoma tumors with increased melanin content are more aggressive and resistant to cancer treatment [55,56,72]. We thus suggest that the increased aggressiveness may be associated with reduced DNA repair with consequent increase in gene mutation. Therefore, *Xpa* was significantly affected by the tumor process, which could ultimately favor the malignization of adjacent tissue due to impaired DNA repair process. Further experiments need to be done to validate this hypothesis.

**Figure 3:**
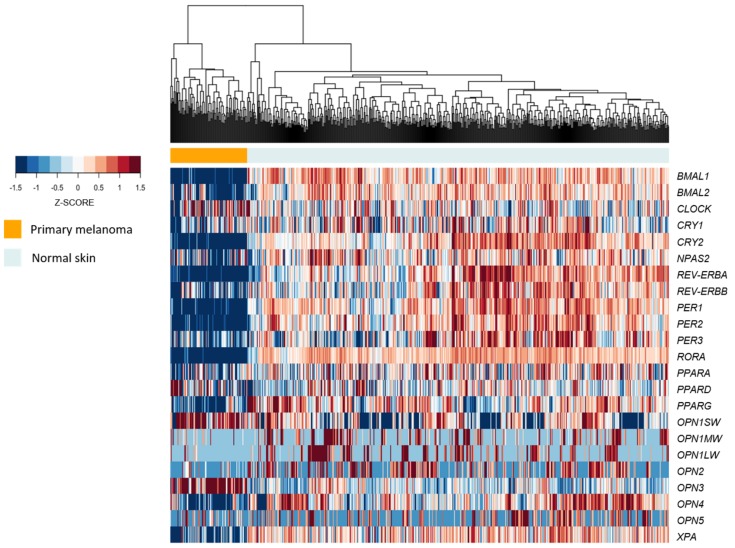
The molecular clock machinery is repressed in human primary melanomas compared to normal skin. Unsupervised hierarchical clustering of 557 GTEx normal (sun and non-sun exposed) skin samples and 104 TCGA primary melanomas according to the expression of clock and clock-controlled genes. Clusters were defined based on the Euclidean distance using complete linkage.

Based on the previous in vivo molecular data, we decided to evaluate the expression of the above-mentioned genes in human samples of healthy skins [73] and primary melanomas [74]. Clinical data analyses show that most of all clock genes, clock-controlled genes, and opsins were less expressed in primary melanoma samples compared to healthy skin. A table containing clinicopathological data from patients with primary melanoma and healthy skin is provided (Supplementary Table S1). In fact, based on the expression levels of these genes, we were able to cluster them in a pattern that clearly distinguishes primary melanoma from healthy skin (Figure 3). Interestingly, primary melanomas show increased *OPN1SW* and *OPN3* expression in comparison to healthy skin, in opposition to all remaining opsins, which are less expressed (Table 2). Therefore, the role of opsins in cancer is an intriguing field that requires further evaluation.

In summary, the presence of tumor and TME has been previously shown to lead to a pro-inflammatory process in adjacent tissue [51], and according to our data, also promotes a chronodisruption in adjacent skin. In addition to the molecular clock, we observed that the expression and/or oscillatory profile of key genes involved in skin proliferation, pigmentation, photo- and thermo-sensitive systems, and DNA repair processes was significantly affected by TME in vivo. The data by Aran and colleagues [51] in association with ours caution against the use of adjacent skin as a “healthy” tissue, since it displays an intermediate phenotype between healthy skin and tumor samples. All these molecular changes clearly demonstrated that tumor-adjacent skin is significantly affected by TME, and that these alterations—especially in the molecular clock—could be linked to the progression of skin cancer.

### 2.2. Effects of Tumor Macroenvironment (TMaE) on Clock and Clock-Controlled Genes of Lung, Liver and SCN

The investigation of the influence of a circumscribed tumor over distant organs is an attractive approach. Although tumors can be confined to a restricted place, even encapsulated, some tumor-derived molecules may be released into the bloodstream. For instance, tumors secrete growth factor-, mRNA- and miRNA-containing micro-vesicles, which can be found in the blood of cancer patients and are important players in the metastatic process (reviewed in [47]). Along this line, a recent study reported that exosomes derived from B16-F10 cells are positive for mRNA and miRNA molecules, which affect the epigenetic landscape and mitochondrial respiration in cytotoxic T cells [75]. Classical effects of TMaE are related to cancer-associated cachexia, systemic inflammation, immune system suppression, and altered coagulation [47,48]. All the previous effects clearly demonstrate the ability of tumors to communicate with the whole organism.

**Figure 4:**
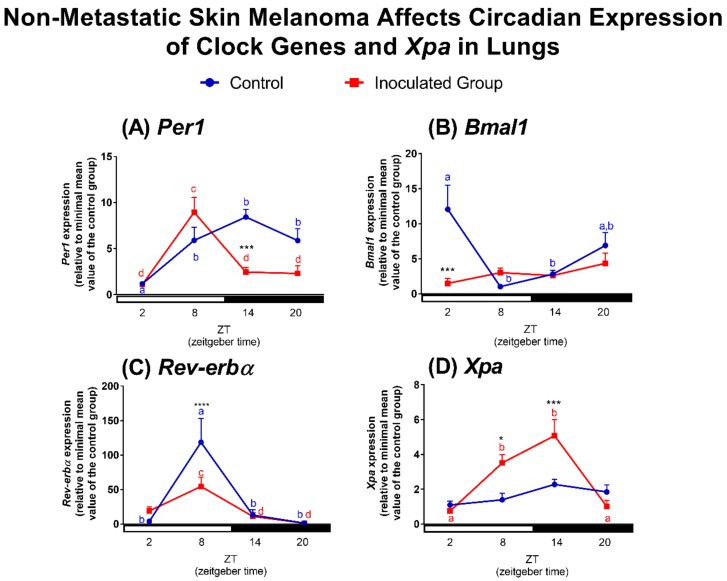
Non-metastatic skin melanoma affects circadian expression of clock genes and *Xpa* in lungs. Expression of (**A**) *Per1*, (**B**) *Bmal1*, (**C**) *Reverb-α* and (**D**) *Xpa* in lungs of control and tumor-bearing mice. Letters represent significant differences in gene expression within the same group, Two-Way ANOVA followed by Bonferroni post-test. In (**A**) *a ≠ b*, *p* < 0.05 and *c ≠ d*, *p* < 0.001. In (**B**) *a ≠ b*, *p* < 0.01. In (**C**) *a ≠ b*, *p* < 0.0001 and *c ≠ d*, *p* < 0.05. In (**D**) *a ≠ b*, *p* < 0.01. Significant differences between control and tumor-bearing mice were demonstrated by Two-Way ANOVA followed by Bonferroni post-test, and are represented by asterisks having the control lung as reference. * *p* <0.05, *** *p* <0.001, **** *p* <0.0001. Values are presented as the mean expression (*n* = 4–6) ± SEM of the gene of interest normalized by 18S RNA, relative to the minimal value found in the control group.

In this study we evaluated two organs commonly affected by melanoma metastasis—lung and liver [76]. In agreement with previous reports of oscillatory profile of clock genes in the lung [77,78,79,80], we showed that lungs of control mice display an oscillatory profile of *Per1* with increased expression at ZTs 8, 14, and 20 in comparison to ZT2. In tumor-bearing mice, lung *Per1* transcripts also exhibited an oscillatory profile, however, with lower amplitude of expression at ZT14 as compared to control mice (Figure 4A). *Bmal1* expression was detected in anti-phase with *Per1* in control animals, with higher level of transcripts at ZT2 in comparison to ZTs 8 and 14 (Figure 4B). In tumor-bearing animals, the circadian oscillation of *Bmal1* was abolished due to a remarkable reduction on transcripts at ZT2 in comparison to control lungs (Figure 4B), and in this case the anti-phase relationship between *Per1* and *Bmal1* was lost. In lungs of control and tumor-bearing mice, *Reverb-α* expression peaked at ZT8, but with a remarkable reduction in amplitude in the tumor-bearing animals (Figure 4C).

It is known that several respiratory parameters such as expiratory flow and lung volume exhibit a circadian pattern [81,82]. Interestingly, this temporal pattern may be altered by oxidative stress evoked by environmental smoke and pollution, jet lag, or pro-inflammatory mediators among others [81]. For instance, nuclear factor (erythroid-derived 2)-like 2 (NRF2), a major antioxidant player, was demonstrated to be a lung CCG and to participate in a time-dependent oxidative/fibrotic lung damage [83]. An elegant study using lung adenocarcinoma demonstrated that jet lag and genetic mutation of *Per2* or *Bmal1* accelerates carcinogenesis with consequent reduction on survival. The lack of clock genes in the lungs resulted in increased c-Myc expression, proliferation, and metabolic dysregulation [84]. Here we demonstrated that the presence of non-metastatic tumor significantly affected the molecular clock of lungs, leading to reduction of *Per1* and *Reverb-α* transcripts and complete loss of *Bmal1* oscillatory pattern, likely resulting in a chronodisruption scenario. Our data suggest that chronodisruption induced by the primary tumor might favor the success of melanoma cells migration and establishment of tumoral environment in the lungs, what still needs to be validated.

In addition to clock genes, we also evaluated *Xpa* expression in lungs of control and tumor-bearing mice. No oscillatory profile was found in lungs of control mice while an oscillatory pattern was found in lungs of tumor-bearing mice: *Xpa* expression was higher at ZTs 8 and 14 than at ZTs 2 and 20, and significantly higher than in control mice (Figure 4D), which could be related to increased DNA damage in this tissue.

**Figure 5:**
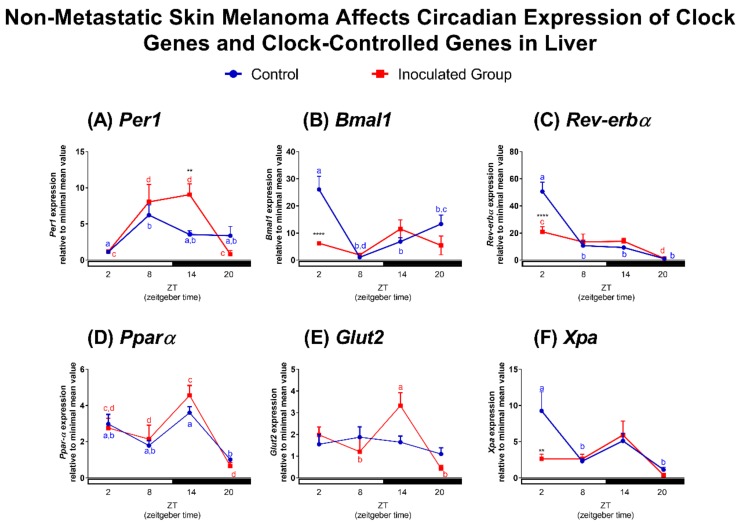
Non-metastatic skin melanoma affects circadian expression of clock genes and clock-controlled genes in liver. Expression of (**A**) *Per1*, (**B**) *Bmal1*, (**C**) *Reverb-α*, (**D**) *Pparα*, (**E**) *Glut2* and (**F**) *Xpa* in liver of control and tumor-bearing mice. Letters represent significant differences in gene expression within the same group, Two-Way ANOVA followed by Bonferroni post-test. In (**A**) *a ≠ b*, *p* < 0.05 and *c ≠ d*, *p* < 0.01. In (**B**) *a ≠ b*, *p* < 0.001, *c ≠ d*, *p* < 0.05. In (**C**) *a ≠ b*, *p* < 0.0001, *c ≠ d*, *p* < 0.05. In (**D**) *a ≠ b* and *c ≠ d*, *p* < 0.05. In (**E**) *a ≠ b*, *p* < 0.05. In (**F**) *a ≠ b*, *p* < 0.05. Significant differences between control and tumor-bearing mice were demonstrated by Two-Way ANOVA followed by Bonferroni post-test, and are represented by asterisks having the control liver as reference ** *p* < 0.01, **** *p* < 0.0001. Values are presented as the mean expression (*n* = 4–6) ± SEM of the gene of interest normalized by 18S RNA, relative to the minimal value found in the control group.

Approximately 10% of all liver transcripts are known to display temporal variation. Among them, several enzymes that participate in cholesterol and lipid metabolism are expressed in a 24-h daily rhythm [85]. Accordingly, it has been shown that plasma triglyceride and cholesterol levels also oscillate along 24 h [86]. In this way, knockout animals of clock components exhibit impairment of lipid metabolism [87,88,89,90] which is related with development of several diseases [4,5,20,21,22], including cancer [13,14,15,17,18,19]. Here we demonstrated, in agreement with the literature reports [78,91,92], that *Per1*, *Bmal1*, and *Reverb-α* display a temporal oscillation in the liver of control mice. *Per1* expression was higher at ZT8 in comparison to the other ZTs; this pattern was conserved in tumor-bearing mice but in these animals *Per1* transcripts remained higher for an additional 6 h (up to ZT14) in comparison to control mice (Figure 5A). We also found an antiphase relationship between liver *Per1* and *Bmal1*. The expression of the latter gene was found to oscillate along 24 h, peaking at ZT2. The oscillatory profile of *Bmal1* was lost in tumor-bearing mice since at ZT2 *Bmal1* expression was significantly reduced when compared to control mice (Figure 5B). The classic antiphase relationship between *Per1* and *Bmal1* observed in the control mice was completely lost in tumor-bearing mice, as a consequence of the shift of *Per1* expression peak and the reduction of *Bmal1* expression and loss of its circadian rhythm. Since *Reverb-α* shows a prominent role in circadian metabolism [93,94], we evaluated its expression in our model. *Reverb-α* exhibited identical oscillatory profile to *Bmal1* gene, with attenuated amplitude at ZT2 in liver of tumor-bearing mice (Figure 5C).

*Pparα*, a CCG, is an important regulator of liver lipid homeostasis since PPARα participates in fatty acid metabolism and ketogenesis [95]. In livers of control animals, *Pparα* expression was higher at ZT14 in comparison to ZT20, a pattern that was still evident in tumor-bearing mice (Figure 5D). We also evaluated the expression of *Glut2*, a gene that encodes a glucose transporter [96,97]. *Glut2* transcripts did not show an oscillatory pattern in control mice; however, in tumor-bearing mice, *Glut2* expression was higher at ZT14 in comparison to ZTs 8 and 20 (Figure 5E). Liver *Xpa* transcripts were higher at ZT2 in comparison to ZTs 8 and 20 in control animals while this oscillatory profile was absent in tumor-bearing mice, as at ZT2, its expression was significantly reduced (Figure 5F). Within this line, Hojo and colleagues [50] found that a non-metastatic breast cancer results in increased oxidative stress in the liver. Due to the similarities in both experimental protocols ([50] and ours), we suggest that liver of tumor-bearing mice may show increased oxidative stress and damage, which could be associated with altered glucose metabolism and DNA repair.

Interestingly, C57BL/6J wild type mice subject to chronic jet lag showed significantly reduced lifespan, spontaneous hepatocellular carcinoma, which is preceded by non-alcoholic fatty liver disease that progress to hepatic steatosis and fibrosis. In addition, these jet-lagged mice had increased hepatic triglycerides and free fatty acids, which was related with insulin resistance [17]. Based on a largescale circadian metabolomics, it was found that most of the analyzed metabolites displayed robust circadian rhythms in control mice; however, jet-lagged mice exhibited a genome-wide gene deregulation and liver metabolic dysfunction with nuclear receptor-controlled cholesterol/bile acids among the top deregulated pathways [17]. In fact, bile acids, besides being a physiological detergent that facilitates intestinal absorption, are also an inflammatory agent that rapidly activates nuclear receptors and cell signaling pathways, which ultimately regulate lipid, glucose, and energy metabolism. The transcriptional regulation of CYP7A1 (cholesterol 7α-hydroxylase), which is the rate limiting enzyme in bile acid biosynthesis, occurs at several levels, mainly related to the levels of plasma cholesterol [98]. In addition to its canonical role, bile acids also stimulate the secretion of pro-inflammatory cytokines from Kupffer cells (liver resident macrophages). This leads to the activation of tumor necrosis factor (TNFα) receptor signaling and mitogen-activated protein kinase (MAPK)/JNK pathway, which results in the repression of *Cyp7a1* [99]. Interestingly, *Reverb-α* negatively regulates *Cyp7a1* by the repression of liver receptor homologue-1 (LRH-1), a known hepatic activator of *Cyp7a1* [100]. In line with the previous data, global or tissue-specific double knockout mice *Rev-erbα^−/−^/Rev-erbβ^−/−^* show elevated plasma triglyceride levels and hepatic steatosis [87,94].

We clearly demonstrated that a non-metastatic melanoma tumor can significantly impair the molecular clock of lungs and liver, indicating a chronodisruption scenario. Taken together the literature and our results, we can hypothesize that TMaE effects on liver and lungs may be due to at least two different mechanisms: (1) change of the temporal profile of clock genes what leads to deregulation of tissue-specific CCGs; (2) activation of immune system that ultimately affects the organ physiology. Within this line, our results open a question whether the altered clock components of distant organs may be a direct or indirect consequence of tumor. In fact, a pioneering study by Masri and colleagues [49] showed that a non-metastatic lung adenocarcinoma rewires, through an inflammatory pathway, several liver clock-controlled metabolic processes, which ultimately impairs insulin, glucose, and lipid metabolism. In similar fashion inoculation of breast cancer cells, in a non-metastatic cancer model, evokes an altered pattern of clock gene expression and increased oxidative stress in liver (not kidney) [50].

Although the results are comparable, important experimental differences should be pointed. Masri and colleagues used a Cre-lox genetic model that leads to activation of the oncogene *Kras* and deletion of *Tp53* (p53-coding gene), and the animals were euthanized 4 months later [49]. Hojo and colleagues [50] used 4T-1 breast cancer cell line subcutaneously injected into mammary gland, and the mice were euthanized 7 days after inoculation. Therefore, comparisons between both studies should be made with caution. The experimental protocol used by Marsi and colleagues [49] mimics a more physiological situation since tumor developed over the course of 4 months. Due to the long experimental setup, the cancer-induced perturbation of the molecular clock may have been compensated, which could explain the lack of alteration of the core clock. On the other hand, Hojo and coworkers [50] used an acute model of tumorigenesis which may have induced an inflammatory process and immune system activation, events that could contribute to the altered profile of the molecular clock profile. Our protocol is similar to the one used by the latter authors, what suggests that the circadian alteration of the clock may be due to the activation of immune system.

**Figure 6:**
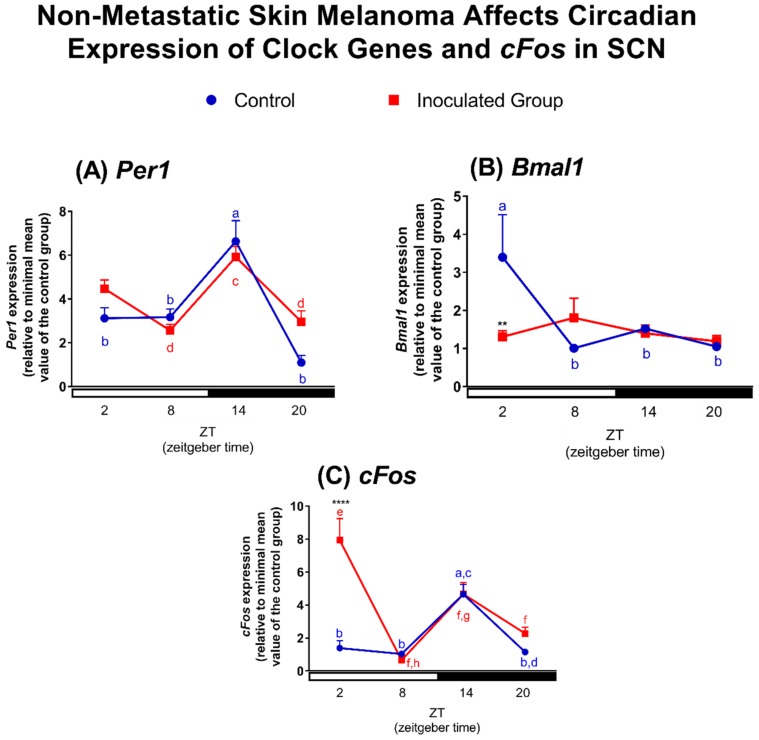
Non-metastatic skin melanoma affects circadian expression of clock genes and *cFos* in SCN. Expression of (**A**) *Per1*, (**B**) *Bmal1* and (**C**) *cFos* in SCN of control and tumor-bearing mice. Letters represent significant differences in gene expression within the same group, Two-Way ANOVA followed by Bonferroni post-test. In (**A**) *a ≠ b*, *p* < 0.01 and *c ≠ d*, *p* < 0.05. In (**B**) *a ≠ b*, *p* < 0.05. In (**C**) *a ≠ b* and *c ≠ d*, *p* < 0.05, *e ≠ f*, *p* < 0.01 and *g ≠ h*, *p* < 0.001. Significant differences between control and tumor-bearing mice were demonstrated by Two-Way ANOVA followed by Bonferroni post-test, and are represented by asterisks having the control SCN as reference. ** *p* < 0.01, **** *p* < 0.0001. Values are presented as the mean expression (*n* = 3–7) ± SEM of the gene of interest normalized by Rpl37a RNA, relative to the minimal value found in the control group.

Based on the fact that lung and liver peripheral clocks were disrupted in tumor-bearing animals, we questioned whether the SCN would still be fully functional in tumor-bearing mice. In SCN of control mice, *Per1* transcripts showed an oscillatory profile with higher levels in the scotophase (ZT14) in comparison to the light phase (ZT2 and ZT8) as well as at ZT20, a pattern that was maintained in tumor-bearing mice (Figure 6A). *Bmal1* transcripts also showed an oscillatory profile in SCN of control mice peaking at ZT2 in antiphase with *Per1*. Surprisingly, in SCN of tumor-bearing mice, the oscillatory profile of *Bmal1* expression, was lost due to a significant transcript reduction at ZT2 in comparison to SCN of control mice (Figure 6B).

We then analyzed *cFos* expression, a gene known to be acutely induced by light, as a marker of neuronal activity [101]. In SCN of control mice, *cFos* expression showed a temporal variation with higher expression at ZT14. In tumor bearing-mice an increased expression of *cFos* was found at ZT2 in comparison to control mice (Figure 6C), which resulted in a phase shift of gene expression phase from ZT14 to ZT2. *cFos* expression was upregulated in tumor-bearing mice compared to control, a fact that could indicate that SCN neuronal activity was increased in response to TMaE, the understanding of which requires additional studies. Therefore, our data clearly show for the first time, that a non-metastatic melanoma impairs the molecular clock function of the central oscillator, mainly due to the reduction of *Bmal1* expression and consequent loss of oscillatory profile and of the antiphase relationship between *Per1* and *Bmal1*.

To the best of our knowledge, no study has evaluated the effect of tumor presence on the molecular clock of the central oscillator. In fact, these are intriguing results since the SCN is known to be resistant to genetic perturbation due to its strong coupling [9,102]. The disruption of the central oscillator is disastrous to the entire organism due to its crucial control of temporal organization [8,12], since important biological processes such as immune system activation [103,104], metabolism [105,106,107], endocrine system physiology [108,109], DNA repair [110] are controlled by the central oscillator in a time-dependent fashion.

It is interesting to highlight that in all analyzed tissues, *Bmal1* disruption paralleled the carcinogenic process. It is known that *Bmal1* is the only clock gene whose removal leads to arrhythmia as no redundant compensatory mechanism exists [12,111,112]. However, BMAL1 protein is regulated by a variety of circadian mechanisms such as phosphorylation, SUMOylation, ubiquitination, acetylation, *O*-GlcNAcylation and *S*-nitrosylation [113]. Additionally, several factors like oxidative stress, nutritional and inflammatory signals significantly affect BMAL1 protein stability and function, thus modifying the organ physiology [113]. Taken altogether, *Bmal1* is an interesting candidate marker for chronodisruption and melanoma development.

Our results bring compelling evidence that a non-metastatic melanoma significantly affects the molecular machinery of both central and peripheral clocks. These data are strengthened by the correlation between tumor melanin content and clock genes. For instance, tumor melanin content negatively correlated with the expression of *Bmal1* and *Per1* in lungs and liver, respectively. Intriguingly, the expression of both genes in SCN samples also negatively correlated with the tumor melanin content. Therefore, as melanin content increases, the expression of clock gene decreases in central and peripheral clocks. Based on these data, we suggest that these clock-related modifications could partially explain the clinical results showing that highly pigmented melanomas are more aggressive than amelanotic ones [55].

We hypothesize that alterations in the clock machinery may be an important step during the onset and progression of the metastatic process as well as could be involved in the development of the classical TMaE alterations such as cachexia, immune system suppression, and altered coagulation. Neither previous reports [49,50] nor the present study evaluated whether the tumor-induced genetic alterations favor tumor metastasis. Further studies are required to establish the consequences of TMaE-induced changes of the molecular clock in cancer development and metastasis. If validated, clock gene and CCG machinery may become an interesting pharmacological target.

The data presented in this manuscript have established important findings regarding the effects of a non-metastatic melanoma on the functioning of central and peripheral clocks. We showed that melanoma tumor exhibits a profound reduction in the expression of clock genes. Of note, melanoma tumors showed an oscillatory profile in melanin content, an event that if proven clinically true, could represent an important treatment strategy since an elevated tumor melanin content has been associated with poor prognosis and limited response to radiotherapy [55]. The presence of tumor is also deleterious to the adjacent tissue, which presented an intermediate phenotype between control skin and tumor, warning against the use of tumor-adjacent tissue as control samples. The TME likely presented alterations in some important skin physiological processes, such as light and temperature detection, proliferation, and DNA repair; however, further functional-based studies are required.

**Figure 7:**
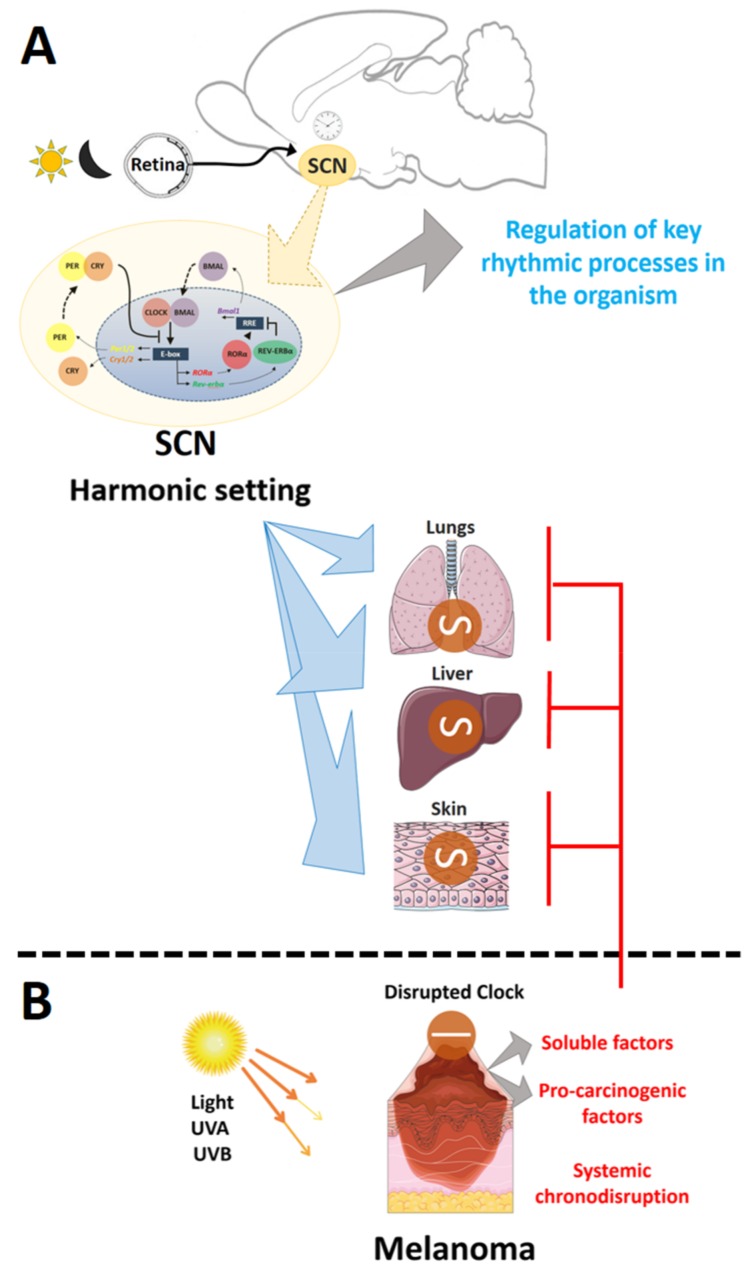
Non-metastatic melanoma leads to systemic chronodisruption in peripheral and central clocks in mice. (**A**) In a physiological situation melanopsin-expressing retinal cells translate the photic information into glutamate release at the suprachiasmatic nucleus (SCN)—the central clock. The SCN then adjusts its molecular clock, comprised by several circadian oscillatory genes, to the environmental light-dark information (24 h duration). The SCN feeds the external time information to several regulatory regions of the brain, which control many biological processes. In fact, in a harmonic setting all biological processes within the organism are in phase and aligned to the external time, thus ensuring a homeostatic condition among all organs and systems; (**B**) However, this systemic harmonic condition is lost when a localized and non-metastatic melanoma is present. Our data show that the effects TME and TMaE lead, respectively, to a chronodisruption scenario in adjacent skin and in distant organs (lungs, liver, and SCN). In addition, TME and TMaE not only disrupt the molecular clock but also affect key tissue-specific regulatory genes. Based on the literature, we suggest that these tumor-induced effects are due to the presence of soluble factors which leak from the encapsulated tumor into the bloodstream, thus triggering systemic chronodisruption.

Regarding TMaE, our data and the cited literature suggest that some tumor-originated molecules may be released into the bloodstream and exert distant effects. In fact, classic alterations of TMaE such as cachexia, systemic inflammation, immune system suppression, and altered coagulation, are well known [47,48]; however, chronodisruption has been recently added to the myriad of TMaE features [49,50]. Within this line, our data provide compelling evidence that chronodisruption, in a non-metastatic model of melanoma, takes place in organs other than the tumor-adjacent skin (Figure 7). These findings, therefore, suggest that chronodisruption could impact melanoma development and progression. The pharmacological targeting of clock components of peripheral tissues might represent an important improvement in oncological treatment [39,114].

## 3. Material and Methods

### 3.1. In Vivo Procedures

Experimental procedures were performed according the protocol approved by the Ethics Committee for Animal Use (CEUA IB/USP, number 255/16, approved on 14 June 2016). The experiments were conducted on 45 C57BL/6J mice (provided by the Institute of Biomedical Sciences vivarium, University of São Paulo, originally acquired from Jackson Laboratories), ranging from eight to sixteen-week old. Animals were subcutaneously inoculated in the right flank with 2 × 10^6^ B16-F10 cells (kindly donated by Prof. Roger Chammas, Faculty of Medicine, University of São Paulo), resuspended in sterile phosphate buffered saline (PBS). Control mice received the same volume of sterile PBS. B16-F10 cells are a metastatic melanoma line capable of forming distant metastasis [115] and the development of metastasis depends on several factors including injection site, number of inoculated cells, and others [115]. In our experimental model no metastasis was observed, and every animal was inspected for the presence of metastasis points in all organs after the euthanasia. Mice were individually placed in standard propylene cages with access to food and water *ad libitum*, kept at 22 °C ± 2 under a 12:12 light/dark cycle (1000–1200 lux white LED light, ranging from 420 to 750 nm). Lights were on at 7 a.m. (ZT0) and off at 7 p.m. The experiments lasted 14 days, and on the 15th day the mice were euthanized with CO_2_ at ZTs 2 (9 a.m.), 8 (3 p.m.), 14 (9 p.m.), and 20 (3 a.m.), and the death was assured by cervical dislocation. At ZTs 14 and 20 all experimental procedures were carried with assistance of night goggles in complete darkness.

The animals were decapitated, the heads wrapped in aluminum foil in the absence of light and placed in dry ice. Then, the ambient light was turned on, and samples of skin (2 cm^2^), liver and lungs from control animals, and of tumor-adjacent skin, tumor, liver, and lungs from melanoma-bearing mice were excised, placed in dry ice, and stored at −80 °C until processing. For SCN isolation, brains were dissected at 4 °C after removal of excess of cranial meninge. Coronal sections were performed using the optic nerves and the base of the posterior hypothalamus as reference. Anterior hypothalamic area was dissected and the SCN was identified by the location of the optic chiasm and third ventricle, and approximately 3 mm thick portion was removed and processed for RNA extraction (Supplementary Figure S1). Because mouse SCN expresses vasoactive intestinal peptide [116], confirmation of *Vip* expression was performed by quantitative PCR.

### 3.2. TME and TMaE Effects in the Organism

To evaluate the effects of TME, we determined gene expression in skin of control mice and tumor-adjacent skin of tumor-bearing mice. The effects of TMaE were assessed by determining the expression of clock genes, clock-controlled genes, and tissue-specific genes in lung, liver, and SCN of control and tumor-inoculated animals.

### 3.3. Total RNA Extraction and Reverse Transcriptase-Polymerase Chain Reaction (RT-PCR)

Small fragments of tissue were homogenized in TRIzol (Thermo Fisher Scientific, Waltham, MA, USA), and total RNA was extracted and purified according to the kit manufacturer’s instructions (Direct-zol™ RNA MiniPrep, Zymo Research, Irvine, CA, USA). RNA concentration (OD_260_) was determined in a spectrophotometer (Nanodrop, Willmington, DE, USA), and 1 μg was subject to reverse transcription with SuperScript III Reverse Transcriptase, random hexamer primers, and other reagents according to the manufacturer’s instructions (Thermo Fisher Scientific, Waltham, MA, USA).

### 3.4. Quantitative PCR (qPCR)

The products of 1 μL of RT-PCR were subject to quantitative PCR reactions using species-specific primers (Table 3) spanning introns, based on sequences obtained from GenBank (http://www.ncbi.nlm.nih.gov/genbank), designed by Primer Blast (http://www.ncbi.nlm.nih.gov/genbank) or Primer Quest (IDT, Coralville, IA, USA), and synthesized by IDT. Rpl37a RNA or ribosomal 18S RNA was used to normalize the expression values of the genes of interest. Prior to this selection, we ascertained that both normalizers did not vary among time points under the various experimental conditions.

For simultaneous analysis of *Per1* and *Bmal1*, multiplex reactions containing cDNA, 300 nM primers, 200 nM fluorescent probes and KAPA PROBE FAST 2× (Kapa Biosystems, Wilmington, MA, USA) were run in triplicates for each experimental cDNA sample. Independent solutions for the remaining genes were prepared with cDNA, specific primers (300 nM) and KAPA SYBR FAST 2× (Kapa Biosystems, Wilmington, MA, USA), and run in duplicates. Reactions were carried out in the following conditions: for multiplex assays, in iQ5 thermocycler (Bio-Rad Laboratories, Hercules, CA, USA), 3 min at 95 °C followed by 45 cycles of 15 s at 95 °C and 60 s at 60 °C; for SYBR Green assays in iQ5 or iCycler thermocycler (Bio-Rad Laboratories, Hercules, CA, USA), 10 min at 95 °C, followed by 45 cycles of 15 s at 95 °C, 1 min at 60 °C, and 80 cycles of 10 s at 55 °C with a gradual rise of 0.5 °C. Negative controls without templates were routinely included.

### 3.5. Melanin Quantification

Melanin content was determined from tumor samples based on a previous study [117]. Briefly, a portion of the tumor was collected, lysed in 1% Triton X-100 in PBS, and kept at 4 °C to allow complete lysis. After vigorous agitation, the samples were centrifuged during 30 min at 14,000× *g* (4 °C) to separate soluble and insoluble fractions. The supernatant was used to quantify total protein by BCA according to the manufacturer’s instruction (Thermo Fisher Scientific, Waltham, MA, USA). The insoluble fraction containing the melanin pellet was resuspended in 1 mL of 1 M NaOH in 10% DMSO and heated at 80 °C during 2 h. Samples were then centrifuged at 1050× *g* for 15 min, the supernatant was transferred to new tubes, and 200 µL of each sample were added in duplicate to wells of a flat-bottom plate. Melanin was quantified by absolute absorbance at 475 nm in a plate reader (SpectraMax 250, Molecular Devices, Sunnyvale, CA, USA), and the values interpolated in a standard curve of synthetic melanin (Sigma-Aldrich, St. Louis, MO, USA) ranging from 3.125 to 200 μg/mL [16,36,37,63]. Values were plotted as µg/mL × 10^3^ previously normalized by protein concentration.

### 3.6. RNAseq Datasets

Gene expression and clinical data of 104 primary melanomas from The Cancer Genoma Atlas (TCGA) and 557 normal skins (sun and non-sun exposed) from Genotype-Tissue Expression (GTEx) were downloaded from the UCSC XENA Browser (http://xena.ucsc.edu) TCGA and GTEx gene expression data were generated using the Illumina HiSeq 2000 RNA sequencing platform, quantified using RSEM, upper quartile normalized and log2(x + 1) transformed. Unsupervised hierarchical clustering was performed using the Euclidean distance and complete linkage. Gene expression data were checked for normality using the D’Agostino & Pearson test. Comparisons between normal skin samples and primary melanomas were performed using the Mann-Whitney test. Analyses were conducted in the R statistical environment (https://www.r-project.org).

### 3.7. Experimental Data Analyses

Gene expression was quantified according the 2^−ΔΔ*C*t^ method [118], as previously described [119]. Δ*C*_t_ was determined by subtracting the normalizer *C*_t_ from the *C*_t_ of the gene of interest at the same time point, both corresponding to the average of duplicate (SybrGreen assays) or triplicate (multiplex assays) wells of the same cDNA. The smaller mean value obtained from control mice was subtracted from all other values, for each tissue for both control and tumor-bearing mice, obtaining the ΔΔ*C*_t_, which was used as a negative exponential of base 2 (2^−ΔΔ*C*t^).

The log values were obtained from at least three animals from two independent experiments. Data are shown as the mean ± SEM. To determine the significance of differences between time points within the same group or the temporal differences between control and tumor-bearing mice, logarithmic data were compared by Two-Way ANOVA followed by Bonferroni or Tukey post-test.

Melanin content of tumor samples was analyzed by One-Way ANOVA followed by Tukey post-test. The analysis of gene correlation with melanin content of matched tumor-bearing mice were carried out using the logarithmic gene expression values for each tissue. Data were checked for normality using the D’Agostino & Pearson test; samples that display a Gaussian distribution were analyzed according to Pearson correlation while non-parametric data were analyzed by Spearman correlation.

In all scenarios, *p* < 0.05 was established to reject the null hypothesis.

## Figures and Tables

**Table 1:** Genes whose expression values correlate with melanin content of matched tumor-bearing mice.

Tissue	Gene Expression vs. Melanin Content	*r* Value	*R*^2^	*p* Value
Tumor-Adjacent Skin	*Per1*	0.4761	0.2266	0.0394
	*Bmal1*	0.6469	0.285	0.0021
	*Opn2*	0.7513	0.5644	0.0003
	*Opn4*	−0.7837	0.09481	0.0001
	*Xpa*	0.5037	0.1475	0.0199
	*Pparγ*	−0.5956	0.3751	0.0133
Tumor	*Per1*	0.5805	0.337	0.0073
	*Bmal1*	0.5651	0.3194	0.0076
	*Opn2*	0.5368	0.2159	0.0147
	*Opn4*	−0.6067	0.368	0.0098
	*Xpa*	−0.5817	0.3383	0.0071
Lungs	*Bmal1*	−0.5015	0.2515	0.0403
Liver	*Per1*	−0.5327	0.1618	0.0356
SCN	*Per1*	0.5543	0.3072	0.0112
	*Bmal1*	−0.5933	0.352	0.0046
	*cFos*	0.6481	0.42	0.002

Positive or negative *r* values indicate positive or negative correlation, respectively, between gene expression in a given tissue and melanin content from tumor samples. Only the genes with significant *p* values are shown. *Mus musculus* gene nomenclature according http://www.informatics.jax.org/mgihome/nomen/gene.shtml.

**Table 2:** Comparison of gene expression between human healthy skins and primary melanomas.

Genes	Expression Mean ± (SEM)		Mann—Whitney Test
	GTEx Normal Skin (*n* = 557)	TCGA Primary Melanoma (*n* = 104)	*p* Value
*BMAL1*	10.525 (0.023)	7.553 (0.08)	**<0.0001**
*BMAL2*	9.45 (0.024)	7.19 (0.183)	**<0.0001**
*CLOCK*	9.975 (0.015)	10.028 (0.079)	**0.019**
*CRY1*	9.623 (0.024)	9.073 (0.089)	**<0.0001**
*CRY2*	12.301 (0.023)	9.737 (0.062)	**<0.0001**
*NPAS2*	11.251 (0.022)	10.346 (0.125)	**<0.0001**
*REV-ERBA*	13.968 (0.051)	10.057 (0.08)	**<0.0001**
*REV-ERBB*	10.826 (0.025)	9.625 (0.111)	**<0.0001**
*PER1*	14.388 (0.033)	10.801 (0.087)	**<0.0001**
*PER2*	11.217 (0.03)	8.404 (0.073)	**<0.0001**
*PER3*	10.842 (0.036)	9.816 (0.089)	**<0.0001**
*RORA*	13.75 (0.031)	7.351 (0.141)	**<0.0001**
*PPARA*	10.24 (0.022)	9.696 (0.079)	**<0.0001**
*PPARD*	11.419 (0.014)	11.525 (0.077)	0.921
*PPARG*	8.08 (0.047)	6.029 (0.135)	**<0.0001**
*OPN1SW*	1.358 (0.047)	2.873 (0.135)	**<0.0001**
*OPN1MW*	0.624 (0.039)	0.219 (0.058)	**<0.0001**
*OPN1LW*	0.628 (0.04)	0.113 (0.039)	**<0.0001**
*OPN2*	0.898 (0.04)	0.29 (0.056)	**<0.0001**
*OPN3*	8.452 (0.027)	10.357 (0.129)	**<0.0001**
*OPN4*	4.709 (0.056)	2.503 (0.149)	**<0.0001**
*OPN5*	0.706 (0.039)	0.554 (0.09)	0.093
*XPA*	8.898 (0.012)	8.002 (0.065)	**<0.0001**

Expression levels were estimated using upper-quartile normalized RSEM. Mean expression is shown as transformed log2(x + 1), where x = RSEM. *p* values in bold indicate significant differences between healthy skin and primary melanoma tumor. *Homo sapiens* gene nomenclature according to https://www.genenames.org/about/guidelines.

**Table 3:** Sequences and concentrations of primers and probes, and gene access numbers.

Templates (Access Number)	Primers and Probes	Final Concentration
*Per1* (NM_0011065.3)	Forward: 5′-AGCAGGTTCAGGCTAACCAGGAAT-3′ Reverse: 5′-AGGTGTCCTGGTTTCGAAGTGTGT-3′ Probe: 5′-/6FAM/-AGCTTGTGCCATGGACATGTCTACT/BHQ_1/-3′	300 nM 300 nM 200 nM
*Bmal1* (NM_001243048)	Forward: 5′-AGCTTCTGCACAATCCACAGCAC-3′ Reverse: 5′-TGTCTGGCTCATTGTCTTCGTCCA-3′ Probe: 5′-/5HEX/-AAAGCTGGCCACCCACGAAGATGGG/BHQ_1/-3′	300 nM 300 nM 200 nM
*Pparγ* (NM_001127330.2)	Forward: 5′-TGTGGGGATAAAGCATCAGGC-3′ Reverse: 5′-CCGGCAGTTAAGATCACACCTAT-3′	300 nM 300 nM
*Pparα* (NM_011144.6)	Forward: 5′-ACGTTTGTGGCTGGTCAAGT-3′ Reverse: 5′-TGGAGAGAGGGTGTCTGTGAT-3′	300 nM 300 nM
*Reverb-α* (NM_145434.4)	Forward: 5′-AAGACATGACGACCCTGGAC-3′ Reverse: 5′-CCATGCCATTCAGCTTGGTAAT-3′	300 nM 300 nM
*Glut2* (NM_031197.2)	Forward: 5′-TGTTGGGGCCATCAACATGA-3′ Reverse: 5′-GGCGAATTTATCCAGCAGCAC-3′	300 nM 300 nM
*Xpa* (NM_011728.2)	Forward: 5′-GGCGATATGAAGCTCTACCTAAA-3′ Reverse: 5′-TTCCTGCCTCACTTCCTTTG-3′	300 nM 300 nM
*cFos (NM_010234.2)*	Forward: 5′-TACTACCATTCCCCAGCCGA-3′ Reverse: 5′-GCTGTCACCGTGGGGATAAA-3′	300 nM 300 nM
*Opn2 (NM_145383.1)*	Forward: 5′-TGCCACACTTGGAGGTGAAA-3′ Reverse: 5′-ACCACGTAGCGCTCAATGG-3′	300 nM 300 nM
*Opn4* (NM_001128599.1)	Forward: 5′-ACATCTTCATCTTCAGGGCCA-3′ Reverse: 5′-ACTCACCGCAGCCCTCAC-3′	300 nM 300 nM
Rpl37a RNA (NM_009084.4)	Forward: 5′-GCATGAAAACAGTGGCCGGT-3′ Reverse: 5′-AGGGTCACACAGTATGTCTCAAAA-3′	300 nM 300 nM
18S RNA	Forward: 5′-CGGCTACCACATCCAAGGAA-3′ Reverse: 5′-GCTGGAATTACCGCGGCT-3′	50 nM 50 nM

## Supplementary Materials

The following are available online at http://www.mdpi.com/1422-0067/19/4/1065/s1.
