# Supplementary material for: Non-Metastatic Cutaneous Melanoma Induces Chronodisruption in Central and Peripheral Circadian Clocks
Source: Int J Mol Sci. 2018 Apr 3;19(4):1065. doi: 10.3390/ijms19041065 (PMC5979525; doi:10.3390/ijms19041065)
Supplement: Supplementary file 1 [file ijms-19-01065-s001.pdf]

Non-Metastatic Cutaneous Melanoma Induces Chronodisruption in Central and Peripheral Circadian Clocks

Leonardo Vinícius Monteiro de Assis [1,†], Maria Nathália Moraes [1,†], Keila Karoline Magalhães-Marques [1], Gabriela Sarti Kinker [2], Sanseray da Silveira Cruz-Machado [2] and Ana Maria de Lauro Castrucci [1,3,*]

[1] Laboratory of Comparative Physiology of Pigmentation, Department of Physiology, Institute of Biosciences, University of São Paulo, São Paulo, 05508-900, Brazil; deassis.leonardo@usp.br (L.V.M.d.A.); nathalia.moraes@usp.br (M.N.M.); keilamagalhaesmarques@gmail.com (K.K.M.-M.)
[2] Laboratory of Chronopharmacology, Department of Physiology, Institute of Biosciences, University of São Paulo, São Paulo, 05508-900, Brazil; gabriela.kinker@hotmail.com (G.S.K.); sanseray@hotmail.com (S.d.S.C.-M.)
[3] Department of Biology, University of Virginia, Charlottesville, VA, 22904, USA
* Correspondence: amdlcast@ib.usp.br; Tel.: +55 11 3091 7523
† These authors contributed equally to this work.

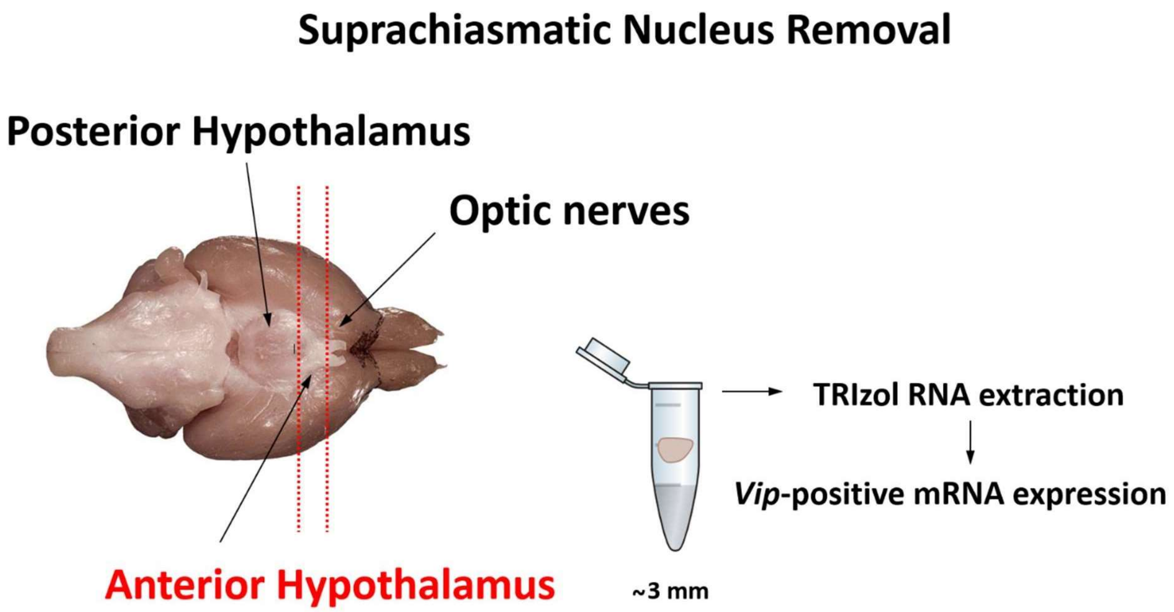

Figure S1. Schematic ventral view of mouse brain. Dashed red lines delineate the area excised from the anterior hypothalamus and that corresponds to the suprachiasmatic nuclei.

Supplementary Table S1 – Clinicopathological characteristics of human normal skin and primary melanoma

		GTEx Normal Skinn = 557 (%)	TCGA Primary Melanoma n = 102 (%)
Gender	Male	365 (66)	35 (34)
	Female	192 (34)	67 (66)
Age	< 60	374 (67)	34 (33)
	≥ 60	183 (33)	68 (67)
Location	Head/neck	0 (0)	8 (8)
	Trunk	232 (42)	47 (46)
	Extremities	325 (58)	41 (40)
Skin color	Other	0 (0)	6 (6)
	Fair	NA	102 (100)
	Medium/dark	NA	0 (0)
